# Supplementary material for: Core Genome Multilocus Sequence Typing for Food Animal Source Attribution of Human Campylobacter jejuni Infections
Source: Pathogens. 2020 Jul 2;9(7):532. doi: 10.3390/pathogens9070532 (PMC7400327; doi:10.3390/pathogens9070532)
Supplement: Supplementary file 1 [file pathogens-09-00532-s001.zip › Campy_source_Sup1_final.pdf]

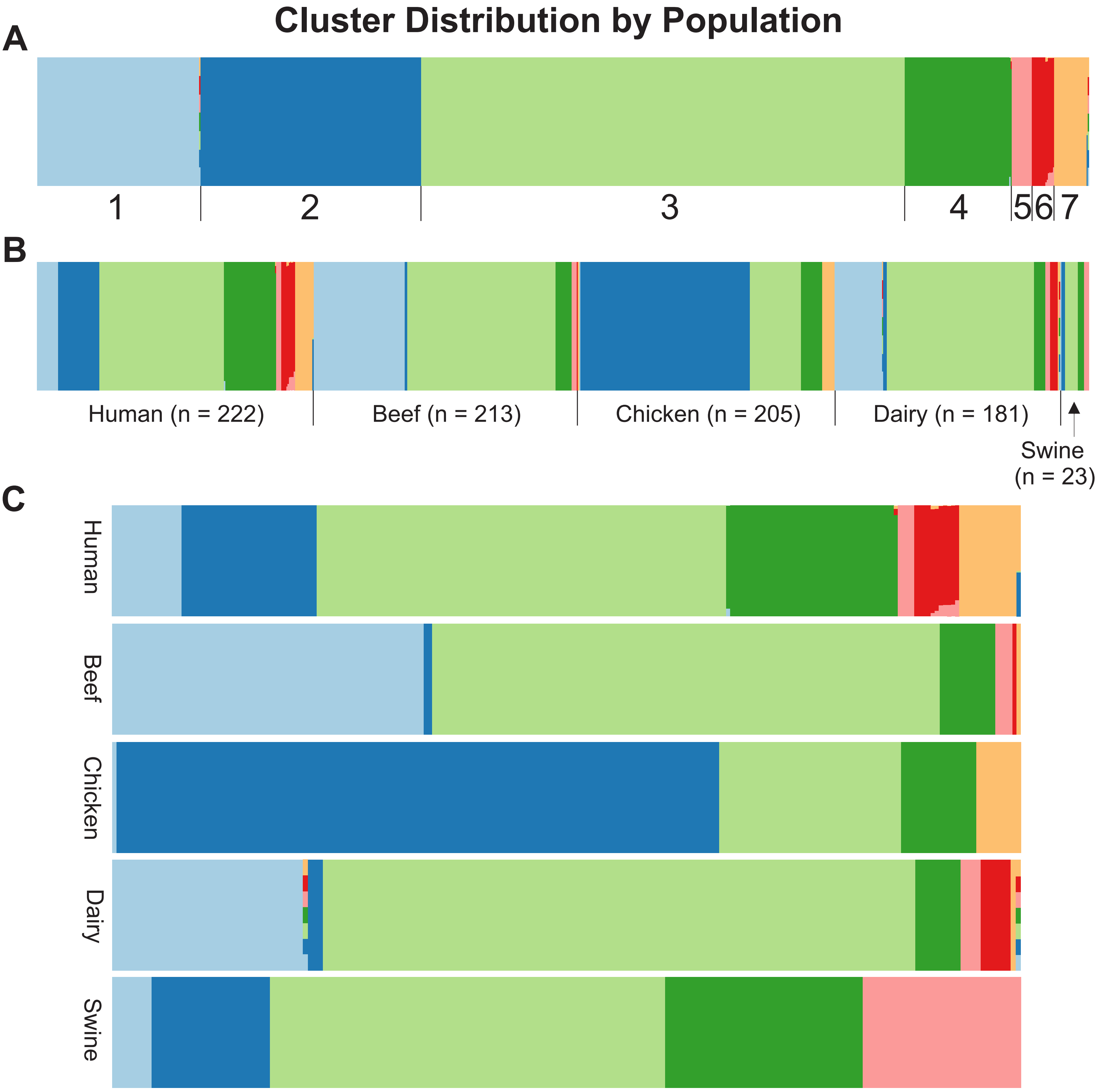


Figure S1: Distribution of conserved population clusters as determined by STRUCTURE analysis of cgMLST allele profiles for 844 *C. jejuni* isolates. Cluster frequencies are shown both by A) aggregate population and B) source animal contribution. C) Proportional contribution of clusters illustrates differences in population composition between animal sources.
