# Supplementary material for: Core Genome Multilocus Sequence Typing for Food Animal Source Attribution of Human Campylobacter jejuni Infections
Source: Pathogens. 2020 Jul 2;9(7):532. doi: 10.3390/pathogens9070532 (PMC7400327; doi:10.3390/pathogens9070532)
Supplement: Supplementary file 1 [file pathogens-09-00532-s001.zip › Campy_source_Sup2_final.pdf]

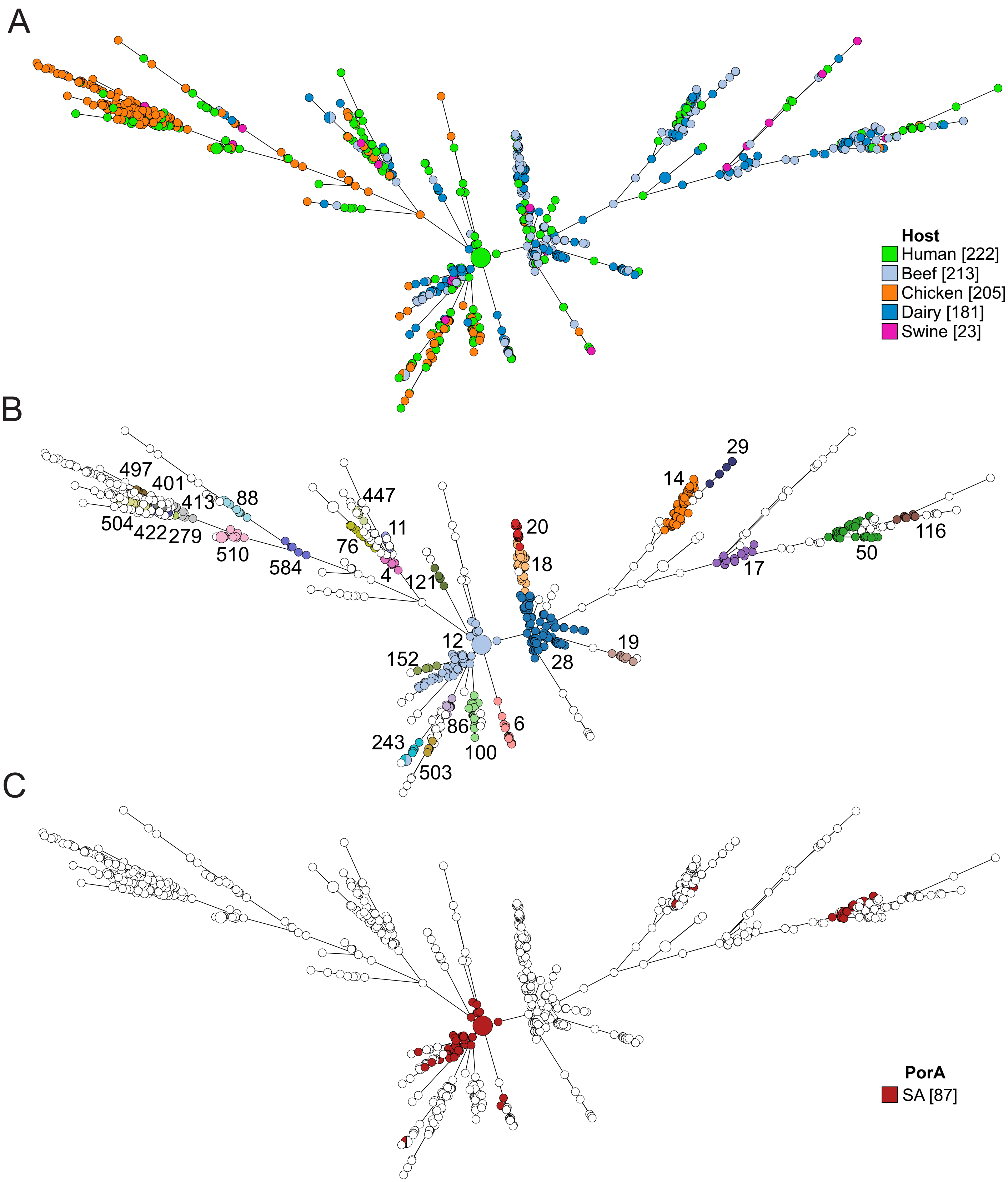

Figure S2: Minimum spanning trees of *C. jejuni* isolates as constructed from cgMLST profiles represented by A) isolation source, B) most populous cgMLST-200 group and C) presence of SA-associated PorA sequence.
