# Supplementary material for: Core Genome Multilocus Sequence Typing for Food Animal Source Attribution of Human Campylobacter jejuni Infections
Source: Pathogens. 2020 Jul 2;9(7):532. doi: 10.3390/pathogens9070532 (PMC7400327; doi:10.3390/pathogens9070532)
Supplement: Supplementary file 1 [file pathogens-09-00532-s001.zip › Campy_source_Sup3_final.pdf]

Supplemental Data

Figure S3:

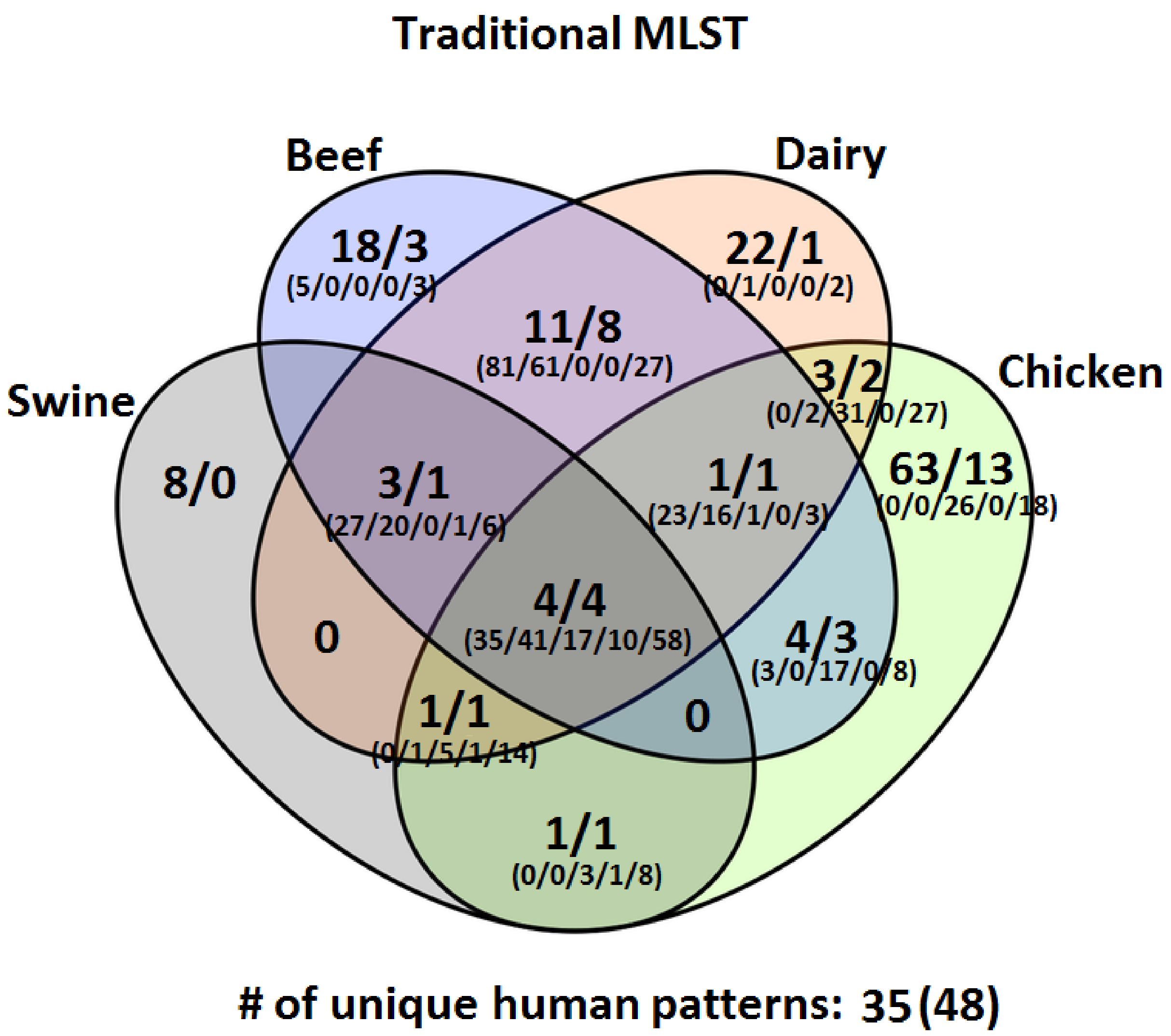

Allelic Difference 200

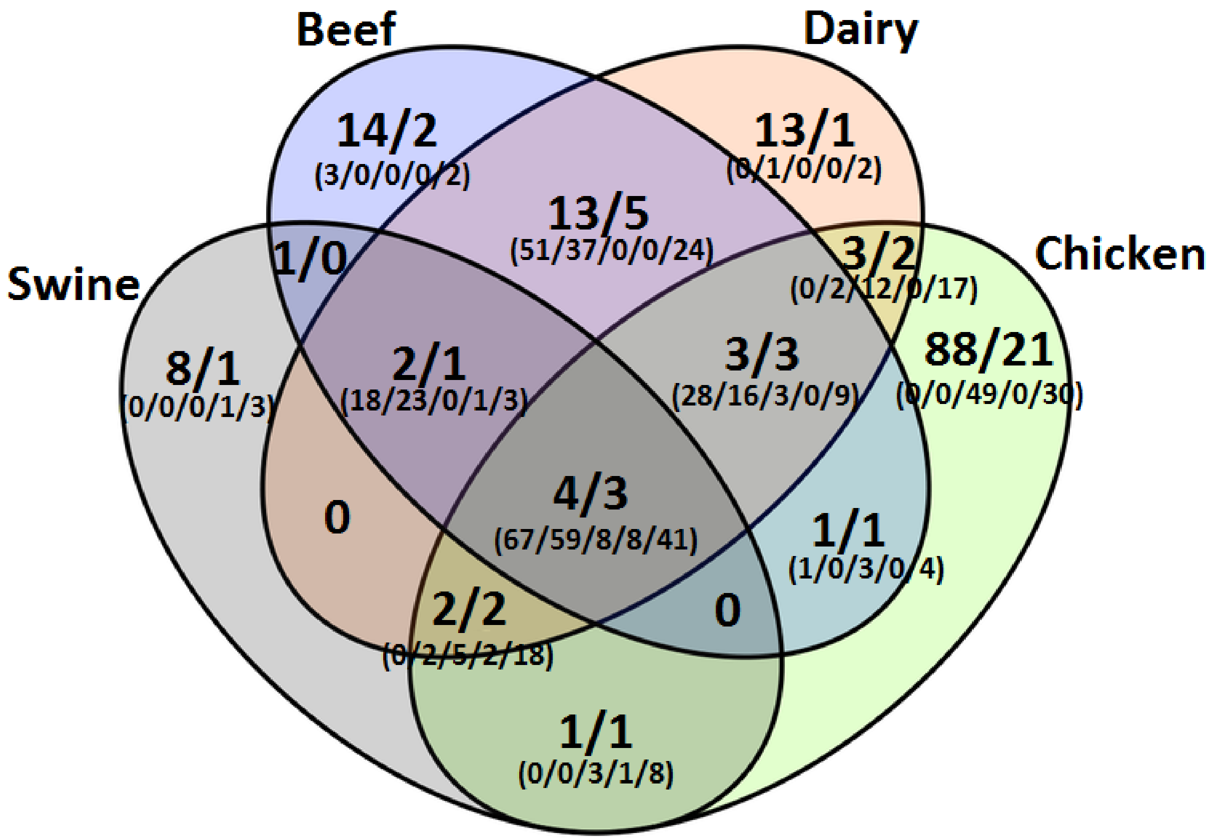

# of unique human patterns: 44 (61)

Allelic Difference 100

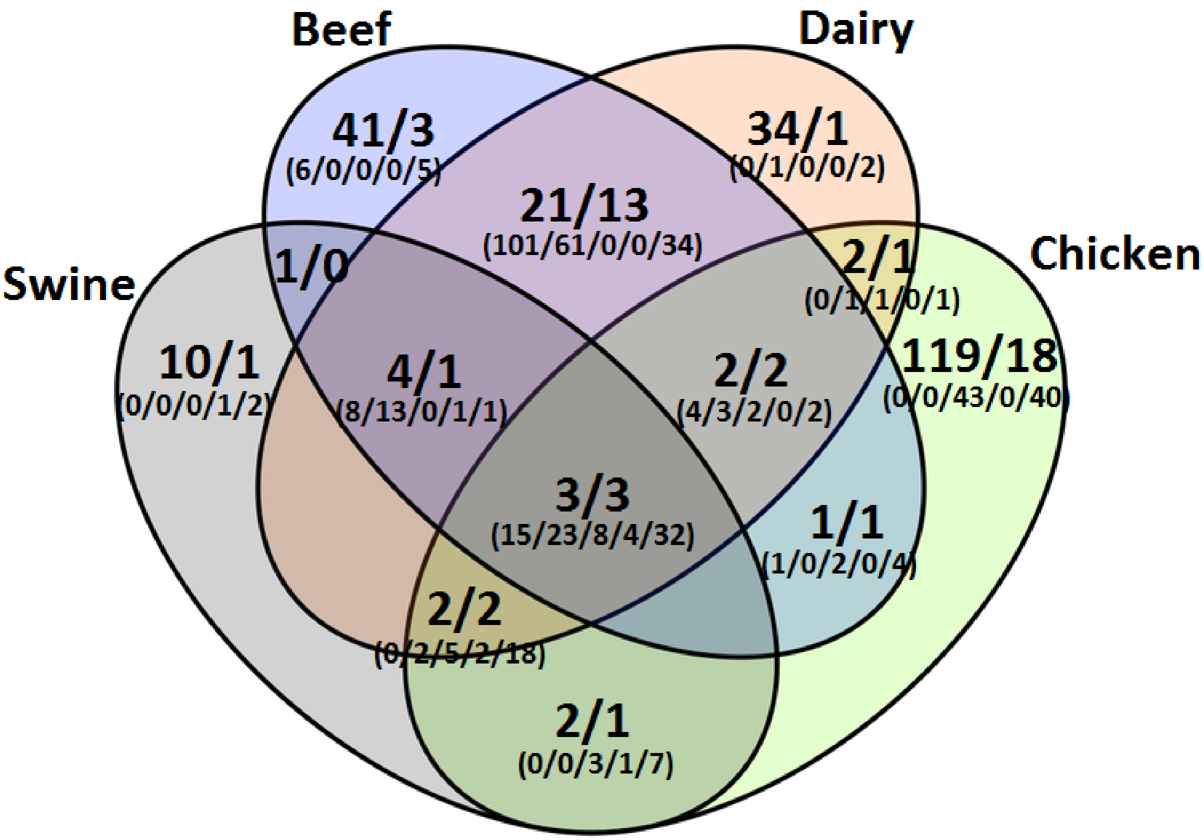

# of unique human patterns: 56 (74)

Allelic Difference 50

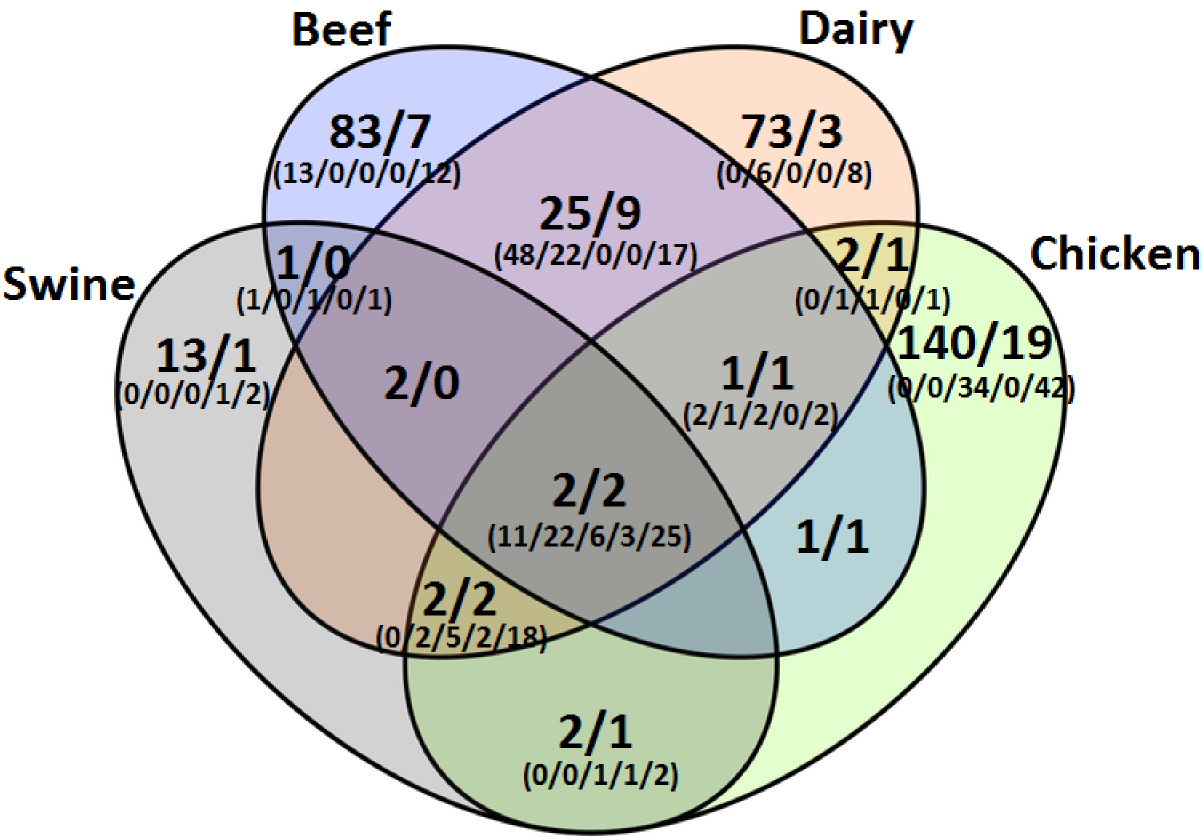

# of unique human patterns: 75(92)

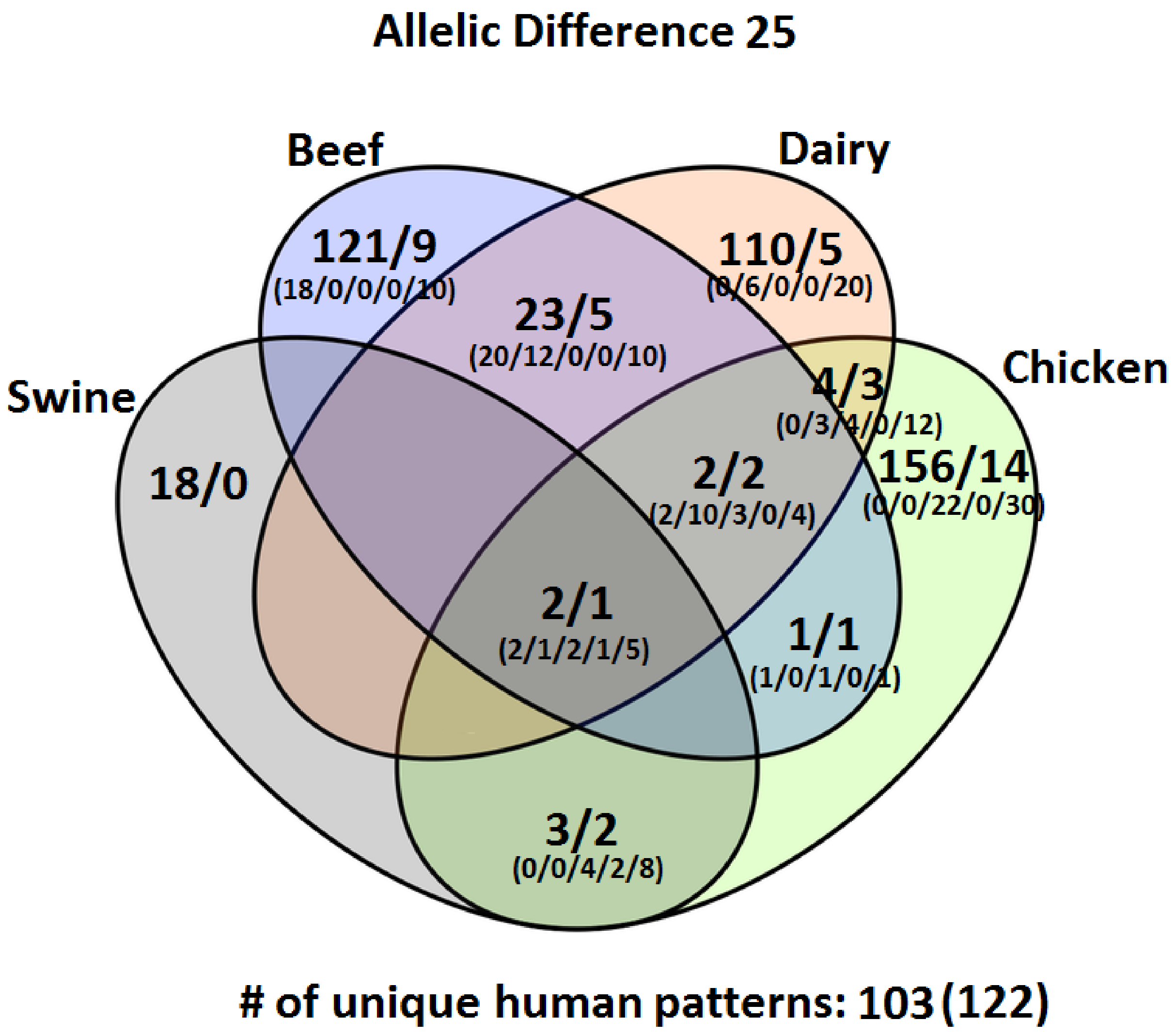
Allelic Difference 25
Beef
Dairy
Swine
Chicken
121/9
(18/0/0/0/10)
110/5
(0/6/0/0/20)
23/5
(20/12/0/0/10)
4/3
(0/3/4/0/12)
18/0
2/2
(2/10/3/0/4)
156/14
(0/0/22/0/30)
2/1
(2/1/2/1/5)
1/1
(1/0/1/0/1)
3/2
(0/0/4/2/8)
# of unique human patterns: 103 (122)

Allelic Difference 10

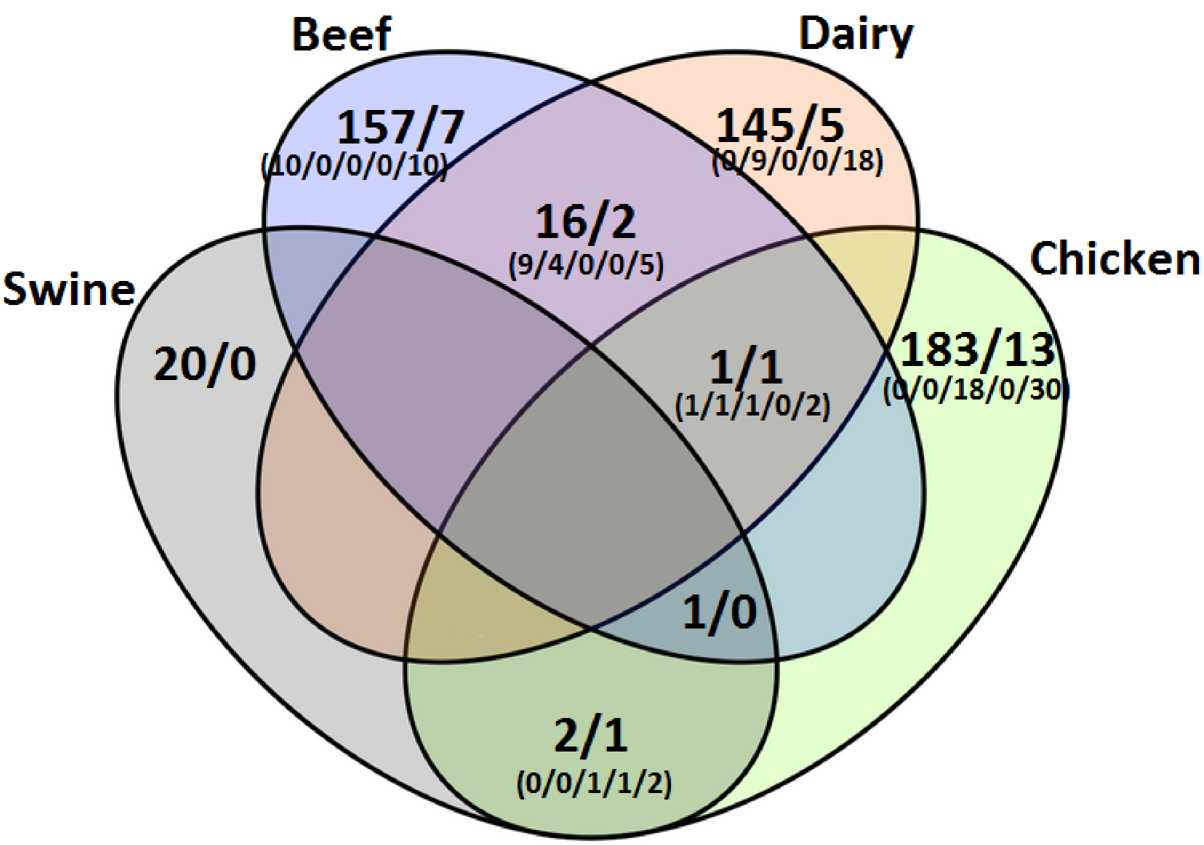

# of unique human patterns: 127 (155)

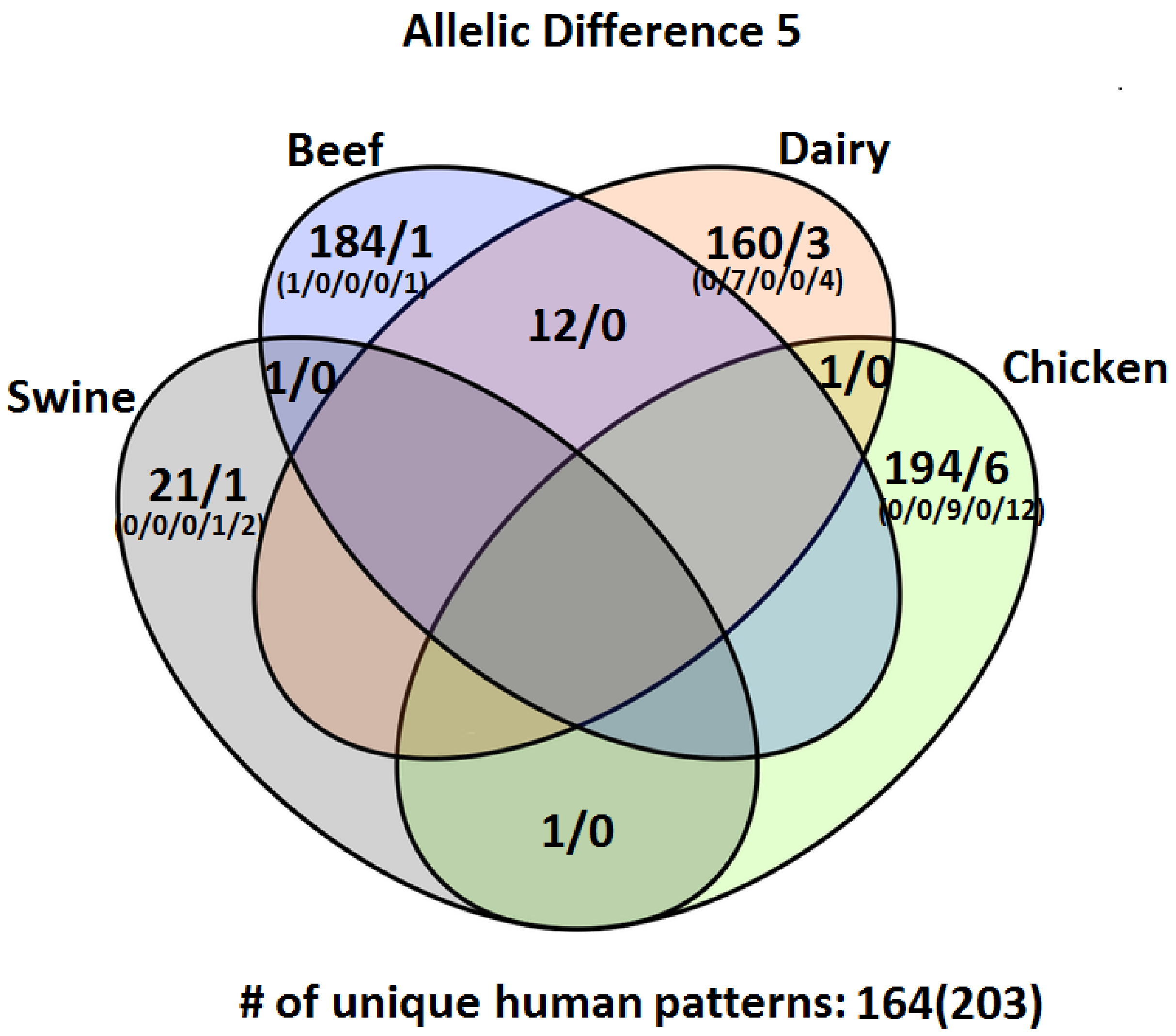

Figure S3. Distribution of sequence type groups (large lettering, above) and isolate composition of human-pathogenic groups (small lettering, below) across multiple food animal sources for different cgMLST thresholds. The total number of groups for each food source is shown to the left of the "/" and the number of groups that contain human pathogenic isolates are on the right. Isolate-level composition of human pathogenic groups below are displayed in the order of "a/b/c/d/e" corresponding to the number of isolates from a) beef cattle, b) dairy cattle, c) chicken, d) swine and e) human.
